# 1-Phenyl-4-(triphenyl­phosphanyl­idene)pyrrolidine-2,3,5-trione

**DOI:** 10.1107/S1600536808003565

**Published:** 2008-02-08

**Authors:** Da-He Fan

**Affiliations:** a School of Chemical and Biological Engineering, Yancheng Institute of Technology, Yancheng 224003, People’s Republic of China

## Abstract

In the title compound, C_28_H_20_NO_3_P, the five-membered maleimide ring is almost planar. The inter­planar angles between the maleimide ring and the three P-bound phenyl rings are 70.6 (2), 60.4 (2) and 54.68 (18)°, while the dihedral angle between the maleimide ring and the N-bound phenyl group is 55.43 (19)°.

## Related literature

For related literature, see: Augustin *et al.* (1979 ▶); Trost & Schmidt (1988 ▶); Mao *et al.* (2005 ▶).
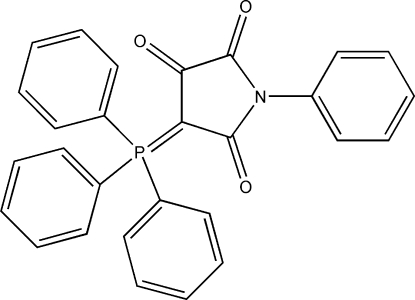


## Experimental

### 

#### Crystal data

C_28_H_20_NO_3_P
*M* *_r_* = 449.42
Orthorhombic, 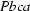

*a* = 18.171 (4) Å
*b* = 12.553 (3) Å
*c* = 19.982 (4) Å
*V* = 4557.9 (16) Å^3^
*Z* = 8
Mo *K*α radiation
μ = 0.15 mm^−1^
*T* = 293 (2) K
0.30 × 0.20 × 0.20 mm

#### Data collection

Enraf–Nonius CAD-4 diffractometer
Absorption correction: ψ scan (*XCAD4*; Harms & Wocadlo, 1995 ▶) *T*_min_ = 0.956, *T*_max_ = 0.970
3961 measured reflections
3961 independent reflections
2590 reflections with *I* > 2σ(*I*)
3 standard reflections every 150 reflections intensity decay: none

#### Refinement

*R*[*F* ^2^ > 2σ(*F* ^2^)] = 0.051
*wR*(*F* ^2^) = 0.180
*S* = 1.07
3961 reflections
299 parameters
H-atom parameters constrained
Δρ_max_ = 0.40 e Å^−3^
Δρ_min_ = −0.34 e Å^−3^

### 

Data collection: *CAD-4 Software* (Enraf–Nonius, 1989 ▶); cell refinement: *CAD-4 Software*; data reduction: *XCAD4* (Harms & Wocadlo, 1995 ▶); program(s) used to solve structure: *SHELXTL* (Sheldrick, 2008 ▶); program(s) used to refine structure: *SHELXTL*; molecular graphics: *SHELXTL*; software used to prepare material for publication: *SHELXTL* and *PLATON* (Spek, 2003 ▶).

## Supplementary Material

Crystal structure: contains datablocks I, global. DOI: 10.1107/S1600536808003565/tk2239sup1.cif

Structure factors: contains datablocks I. DOI: 10.1107/S1600536808003565/tk2239Isup2.hkl

Additional supplementary materials:  crystallographic information; 3D view; checkCIF report

## Figures and Tables

**Fig. 1.**
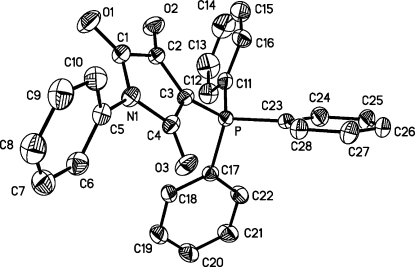
The molecular structure of (I) showing 30% probability displacement ellipsoids and the atom labelling scheme.

## supplementary crystallographic information

### Comment

Organophosphorus compounds play an important role in organic synthesis. In some reactions, a nucleophilic tertiary phosphine initially adds to the triple bond of an electron-deficient alkyne and is finally eliminated from the reaction product after a series of transformations. As such, the tertiary phosphine plays the role of a catalyst (Trost *et al.*, 1988). In recent experiments, the title compound, (I), was synthesized unexpectedly when triphenylphosphine, Ph_3_P, was used as a catalyst in a reaction with *N*-phenylmaleimide (Augustin *et al.*, 1979). In this reaction, the Ph_3_P was not eliminated but instead reacted (Mao *et al.*, 2005). The stucture of (I), Fig. 1, shows the interplanar angles between the maleimide ring and the three P-bound phenyl rings to be 70.6 (2), 60.4 (2) and 54.68 (18)°, respectively, and the dihedral angle between the maleimide ring and the N-bound phenyl group to be 55.4 (3)°.

### Experimental

To a CH_2_Cl_2_ (5 ml) solution comprising Ph_3_P (0.06 g, 0.00014 mmol), *N*-phenylmaleimide (0.173 g, 1.0 mmol) and 1-aminobenzotriazole (0.201 g, 1.5 mmol), lead tetraacetate (0.666 g, 1.5 mmol) in CH_2_Cl_2_ (5 ml) was added dropwise. After stirring at room temperature for 30 min, the solution was concentrated and separated by flash chromatography on a silica gel column with petroleum ether/ethyl acetate as eluent. Compound (I) was isolated as one of the products (yield 2.4%); m. pt. 526 K.

### Refinement

The H atoms were fixed geometrically and were treated as riding on their parent C atoms, with C—H = 0.93 Å, and with *U*_iso_(H) = 1.2*U*_eq_(C).

### Crystal data

**Table:**

C_28_H_20_NO_3_P	*D*_x_ = 1.310 Mg m^−^^3^
*M**_r_* = 449.42	Melting point: 527 K
Orthorhombic, *P**b**c**a*	Mo *K*α radiation λ = 0.71073 Å
Hall symbol: -P 2ac 2ab	Cell parameters from 187 reflections
*a* = 18.171 (4) Å	θ = 3.6–45.0º
*b* = 12.553 (3) Å	µ = 0.15 mm^−^^1^
*c* = 19.982 (4) Å	*T* = 293 (2) K
*V* = 4557.9 (16) Å^3^	Block, colorless
*Z* = 8	0.30 × 0.20 × 0.20 mm
*F*_000_ = 1872	

### Data collection

**Table:**

Enraf–Nonius CAD-4 diffractometer	*R*_int_ = 0.000
Radiation source: fine-focus sealed tube	θ_max_ = 25.0º
Monochromator: graphite	θ_min_ = 2.0º
*T* = 293(2) K	*h* = 0→21
ω/2θ scans	*k* = 0→14
Absorption correction: ψ scan(XCAD4; Harms & Wocadlo, 1995)	*l* = −23→0
*T*_min_ = 0.956, *T*_max_ = 0.970	3 standard reflections
3961 measured reflections	every 150 reflections
3961 independent reflections	intensity decay: none
2590 reflections with *I* > 2σ(*I*)	

### Refinement

**Table:**

Refinement on *F*^2^	Hydrogen site location: inferred from neighbouring sites
Least-squares matrix: full	H-atom parameters constrained
*R*[*F*^2^ > 2σ(*F*^2^)] = 0.051	*w* = 1/[σ^2^(*F*_o_^2^) + (0.0776*P*)^2^ + 4.6891*P*] where *P* = (*F*_o_^2^ + 2*F*_c_^2^)/3
*wR*(*F*^2^) = 0.180	(Δ/σ)_max_ < 0.001
*S* = 1.07	Δρ_max_ = 0.40 e Å^−^^3^
3961 reflections	Δρ_min_ = −0.34 e Å^−^^3^
299 parameters	Extinction correction: SHELXTL (Sheldrick, 2008), Fc^*^=kFc[1+0.001xFc^2^λ^3^/sin(2θ)]^-1/4^
Primary atom site location: structure-invariant direct methods	Extinction coefficient: 0.0079 (7)
Secondary atom site location: difference Fourier map	

### Special details

**Table:**

Geometry. All e.s.d.'s (except the e.s.d. in the dihedral angle between two l.s. planes) are estimated using the full covariance matrix. The cell e.s.d.'s are taken into account individually in the estimation of e.s.d.'s in distances, angles and torsion angles; correlations between e.s.d.'s in cell parameters are only used when they are defined by crystal symmetry. An approximate (isotropic) treatment of cell e.s.d.'s is used for estimating e.s.d.'s involving l.s. planes.
Refinement. Refinement of *F*^2^ against ALL reflections. The weighted *R*-factor *wR* and goodness of fit *S* are based on *F*^2^, conventional *R*-factors *R* are based on *F*, with *F* set to zero for negative *F*^2^. The threshold expression of *F*^2^ > σ(*F*^2^) is used only for calculating *R*-factors(gt) *etc*. and is not relevant to the choice of reflections for refinement. *R*-factors based on *F*^2^ are statistically about twice as large as those based on *F*, and *R*- factors based on ALL data will be even larger.

### Fractional atomic coordinates and isotropic or equivalent isotropic displacement parameters (Å^2^)

**Table:**

	*x*	*y*	*z*	*U*_iso_*/*U*_eq_	
C1	0.14772 (19)	0.5354 (3)	0.47338 (17)	0.0463 (9)	
C2	0.14220 (18)	0.6388 (3)	0.43154 (16)	0.0420 (8)	
C3	0.10163 (18)	0.6110 (3)	0.37341 (15)	0.0377 (8)	
C4	0.07940 (18)	0.5005 (3)	0.37848 (16)	0.0408 (8)	
C5	0.0969 (2)	0.3513 (3)	0.46274 (16)	0.0436 (8)	
C6	0.0270 (2)	0.3128 (3)	0.46980 (19)	0.0532 (10)	
H6A	−0.0131	0.3545	0.4577	0.064*	
C7	0.0162 (3)	0.2110 (3)	0.4952 (2)	0.0619 (11)	
H7A	−0.0313	0.1845	0.5003	0.074*	
C8	0.0757 (3)	0.1496 (3)	0.5128 (2)	0.0647 (11)	
H8A	0.0686	0.0816	0.5301	0.078*	
C9	0.1461 (3)	0.1888 (3)	0.5048 (2)	0.0645 (11)	
H9A	0.1863	0.1470	0.5166	0.077*	
C10	0.1572 (2)	0.2895 (3)	0.47953 (19)	0.0516 (9)	
H10A	0.2046	0.3157	0.4738	0.062*	
C11	0.1025 (2)	0.8272 (3)	0.32675 (17)	0.0448 (8)	
C12	0.0544 (3)	0.9069 (3)	0.3436 (2)	0.0627 (11)	
H12A	0.0041	0.8933	0.3448	0.075*	
C13	0.0807 (4)	1.0078 (4)	0.3588 (3)	0.0909 (17)	
H13A	0.0483	1.0624	0.3696	0.109*	
C14	0.1563 (4)	1.0261 (4)	0.3575 (3)	0.0956 (18)	
H14A	0.1744	1.0932	0.3685	0.115*	
C15	0.2036 (3)	0.9482 (4)	0.3407 (2)	0.0782 (14)	
H15A	0.2538	0.9621	0.3394	0.094*	
C16	0.1779 (2)	0.8478 (4)	0.3253 (2)	0.0607 (11)	
H16A	0.2108	0.7939	0.3140	0.073*	
C17	−0.02614 (17)	0.6940 (3)	0.30122 (16)	0.0388 (8)	
C18	−0.07044 (19)	0.6465 (3)	0.34892 (18)	0.0513 (9)	
H18A	−0.0492	0.6132	0.3858	0.062*	
C19	−0.1459 (2)	0.6482 (4)	0.3422 (2)	0.0695 (13)	
H19A	−0.1755	0.6137	0.3733	0.083*	
C20	−0.1774 (2)	0.7013 (4)	0.2891 (2)	0.0678 (12)	
H20A	−0.2284	0.7045	0.2854	0.081*	
C21	−0.1342 (2)	0.7497 (4)	0.2415 (2)	0.0635 (11)	
H21A	−0.1560	0.7850	0.2057	0.076*	
C22	−0.05895 (19)	0.7460 (3)	0.24670 (19)	0.0520 (9)	
H22A	−0.0297	0.7779	0.2142	0.062*	
C23	0.11017 (17)	0.6596 (3)	0.22757 (16)	0.0404 (8)	
C24	0.1195 (2)	0.7381 (4)	0.17908 (19)	0.0568 (10)	
H24A	0.1071	0.8084	0.1885	0.068*	
C25	0.1475 (2)	0.7109 (4)	0.11636 (19)	0.0642 (12)	
H25A	0.1540	0.7631	0.0839	0.077*	
C26	0.1653 (2)	0.6068 (5)	0.1026 (2)	0.0694 (13)	
H26A	0.1839	0.5886	0.0607	0.083*	
C27	0.1559 (2)	0.5302 (4)	0.1501 (2)	0.0667 (12)	
H27A	0.1681	0.4600	0.1403	0.080*	
C28	0.1284 (2)	0.5556 (3)	0.21299 (19)	0.0536 (10)	
H28A	0.1224	0.5027	0.2451	0.064*	
N1	0.10879 (16)	0.4592 (2)	0.43984 (14)	0.0445 (7)	
O1	0.17921 (16)	0.5257 (2)	0.52663 (13)	0.0717 (9)	
O2	0.16942 (15)	0.7217 (2)	0.45206 (13)	0.0610 (8)	
O3	0.04234 (15)	0.4470 (2)	0.34108 (12)	0.0568 (7)	
P	0.07209 (4)	0.69405 (7)	0.30865 (4)	0.0358 (3)	

### Atomic displacement parameters (Å^2^)

**Table:**

	*U*^11^	*U*^22^	*U*^33^	*U*^12^	*U*^13^	*U*^23^
C1	0.0449 (19)	0.056 (2)	0.0382 (19)	0.0003 (17)	−0.0043 (16)	−0.0005 (17)
C2	0.0376 (17)	0.052 (2)	0.0361 (18)	0.0013 (16)	−0.0016 (15)	−0.0003 (16)
C3	0.0375 (17)	0.0408 (19)	0.0348 (17)	0.0025 (15)	−0.0036 (14)	−0.0018 (14)
C4	0.0430 (19)	0.045 (2)	0.0341 (17)	0.0074 (16)	−0.0041 (15)	−0.0012 (15)
C5	0.051 (2)	0.045 (2)	0.0353 (18)	0.0036 (17)	0.0005 (15)	0.0006 (15)
C6	0.053 (2)	0.055 (2)	0.052 (2)	0.0084 (19)	−0.0034 (18)	0.0045 (18)
C7	0.071 (3)	0.056 (3)	0.059 (2)	−0.010 (2)	0.000 (2)	0.007 (2)
C8	0.086 (3)	0.044 (2)	0.064 (3)	0.005 (2)	0.001 (2)	0.009 (2)
C9	0.077 (3)	0.056 (3)	0.061 (3)	0.021 (2)	0.001 (2)	0.010 (2)
C10	0.048 (2)	0.053 (2)	0.054 (2)	0.0111 (17)	0.0017 (17)	0.0042 (18)
C11	0.049 (2)	0.048 (2)	0.0376 (18)	−0.0043 (17)	0.0016 (15)	0.0031 (15)
C12	0.073 (3)	0.050 (2)	0.064 (3)	0.005 (2)	0.004 (2)	−0.007 (2)
C13	0.120 (5)	0.050 (3)	0.102 (4)	0.004 (3)	0.016 (3)	−0.016 (3)
C14	0.141 (6)	0.056 (3)	0.090 (4)	−0.035 (4)	−0.009 (4)	−0.009 (3)
C15	0.089 (4)	0.077 (3)	0.069 (3)	−0.040 (3)	0.004 (3)	−0.004 (3)
C16	0.053 (2)	0.065 (3)	0.064 (3)	−0.015 (2)	0.0049 (19)	−0.003 (2)
C17	0.0333 (17)	0.0425 (18)	0.0404 (18)	0.0039 (15)	−0.0031 (14)	−0.0014 (15)
C18	0.0388 (19)	0.075 (3)	0.0404 (19)	−0.0013 (18)	0.0010 (16)	0.0088 (18)
C19	0.043 (2)	0.108 (4)	0.057 (2)	−0.007 (2)	0.0007 (19)	0.017 (2)
C20	0.033 (2)	0.100 (4)	0.070 (3)	0.004 (2)	0.0008 (19)	0.012 (3)
C21	0.042 (2)	0.077 (3)	0.072 (3)	0.003 (2)	−0.010 (2)	0.021 (2)
C22	0.0396 (19)	0.062 (2)	0.055 (2)	0.0029 (18)	−0.0015 (17)	0.0202 (19)
C23	0.0318 (16)	0.056 (2)	0.0334 (17)	−0.0008 (16)	−0.0025 (14)	−0.0026 (15)
C24	0.049 (2)	0.074 (3)	0.047 (2)	0.006 (2)	−0.0005 (17)	0.009 (2)
C25	0.053 (2)	0.106 (4)	0.034 (2)	0.004 (2)	−0.0038 (17)	0.012 (2)
C26	0.052 (2)	0.118 (4)	0.038 (2)	−0.009 (3)	0.0076 (18)	−0.018 (3)
C27	0.069 (3)	0.081 (3)	0.050 (2)	−0.002 (2)	0.008 (2)	−0.025 (2)
C28	0.053 (2)	0.065 (3)	0.043 (2)	−0.0003 (19)	0.0045 (17)	−0.0066 (18)
N1	0.0512 (17)	0.0441 (17)	0.0382 (15)	0.0040 (14)	−0.0062 (13)	0.0036 (13)
O1	0.087 (2)	0.075 (2)	0.0529 (17)	−0.0132 (17)	−0.0312 (16)	0.0138 (14)
O2	0.0692 (18)	0.0578 (17)	0.0560 (16)	−0.0164 (14)	−0.0168 (14)	−0.0007 (13)
O3	0.0769 (18)	0.0463 (15)	0.0472 (15)	−0.0062 (13)	−0.0173 (14)	−0.0067 (12)
P	0.0330 (4)	0.0412 (5)	0.0331 (5)	0.0014 (4)	0.0001 (3)	0.0001 (4)

### Geometric parameters (Å, °)

**Table:**

C1—O1	1.214 (4)	C14—H14A	0.9300
C1—N1	1.365 (4)	C15—C16	1.379 (6)
C1—C2	1.547 (5)	C15—H15A	0.9300
C2—O2	1.223 (4)	C16—H16A	0.9300
C2—C3	1.419 (4)	C17—C18	1.383 (5)
C3—C4	1.448 (5)	C17—C22	1.403 (5)
C3—P	1.746 (3)	C17—P	1.791 (3)
C4—O3	1.210 (4)	C18—C19	1.379 (5)
C4—N1	1.434 (4)	C18—H18A	0.9300
C5—C6	1.367 (5)	C19—C20	1.377 (6)
C5—C10	1.383 (5)	C19—H19A	0.9300
C5—N1	1.447 (4)	C20—C21	1.376 (6)
C6—C7	1.388 (5)	C20—H20A	0.9300
C6—H6A	0.9300	C21—C22	1.372 (5)
C7—C8	1.375 (6)	C21—H21A	0.9300
C7—H7A	0.9300	C22—H22A	0.9300
C8—C9	1.380 (6)	C23—C28	1.377 (5)
C8—H8A	0.9300	C23—C24	1.393 (5)
C9—C10	1.376 (6)	C23—P	1.814 (3)
C9—H9A	0.9300	C24—C25	1.395 (5)
C10—H10A	0.9300	C24—H24A	0.9300
C11—C12	1.371 (5)	C25—C26	1.374 (6)
C11—C16	1.394 (5)	C25—H25A	0.9300
C11—P	1.798 (4)	C26—C27	1.362 (6)
C12—C13	1.387 (6)	C26—H26A	0.9300
C12—H12A	0.9300	C27—C28	1.389 (5)
C13—C14	1.395 (8)	C27—H27A	0.9300
C13—H13A	0.9300	C28—H28A	0.9300
C14—C15	1.344 (8)		
			
O1—C1—N1	127.1 (4)	C11—C16—H16A	120.1
O1—C1—C2	126.1 (3)	C18—C17—C22	119.2 (3)
N1—C1—C2	106.8 (3)	C18—C17—P	121.6 (3)
O2—C2—C3	133.9 (3)	C22—C17—P	119.2 (3)
O2—C2—C1	120.4 (3)	C19—C18—C17	120.3 (4)
C3—C2—C1	105.7 (3)	C19—C18—H18A	119.8
C2—C3—C4	108.8 (3)	C17—C18—H18A	119.8
C2—C3—P	128.3 (3)	C20—C19—C18	119.8 (4)
C4—C3—P	122.5 (2)	C20—C19—H19A	120.1
O3—C4—N1	122.3 (3)	C18—C19—H19A	120.1
O3—C4—C3	130.1 (3)	C21—C20—C19	120.6 (4)
N1—C4—C3	107.6 (3)	C21—C20—H20A	119.7
C6—C5—C10	120.9 (3)	C19—C20—H20A	119.7
C6—C5—N1	120.2 (3)	C22—C21—C20	120.1 (4)
C10—C5—N1	118.9 (3)	C22—C21—H21A	120.0
C5—C6—C7	119.6 (4)	C20—C21—H21A	120.0
C5—C6—H6A	120.2	C21—C22—C17	119.9 (3)
C7—C6—H6A	120.2	C21—C22—H22A	120.0
C8—C7—C6	120.0 (4)	C17—C22—H22A	120.0
C8—C7—H7A	120.0	C28—C23—C24	119.6 (3)
C6—C7—H7A	120.0	C28—C23—P	120.5 (3)
C7—C8—C9	119.9 (4)	C24—C23—P	119.9 (3)
C7—C8—H8A	120.0	C23—C24—C25	119.7 (4)
C9—C8—H8A	120.0	C23—C24—H24A	120.1
C10—C9—C8	120.4 (4)	C25—C24—H24A	120.1
C10—C9—H9A	119.8	C26—C25—C24	120.0 (4)
C8—C9—H9A	119.8	C26—C25—H25A	120.0
C9—C10—C5	119.2 (4)	C24—C25—H25A	120.0
C9—C10—H10A	120.4	C27—C26—C25	120.1 (4)
C5—C10—H10A	120.4	C27—C26—H26A	120.0
C12—C11—C16	119.8 (4)	C25—C26—H26A	120.0
C12—C11—P	122.1 (3)	C26—C27—C28	121.0 (4)
C16—C11—P	118.0 (3)	C26—C27—H27A	119.5
C11—C12—C13	120.0 (5)	C28—C27—H27A	119.5
C11—C12—H12A	120.0	C23—C28—C27	119.7 (4)
C13—C12—H12A	120.0	C23—C28—H28A	120.2
C12—C13—C14	119.1 (5)	C27—C28—H28A	120.2
C12—C13—H13A	120.4	C1—N1—C4	111.1 (3)
C14—C13—H13A	120.4	C1—N1—C5	125.3 (3)
C15—C14—C13	120.9 (5)	C4—N1—C5	123.6 (3)
C15—C14—H14A	119.5	C3—P—C17	111.55 (16)
C13—C14—H14A	119.5	C3—P—C11	108.15 (16)
C14—C15—C16	120.3 (5)	C17—P—C11	108.89 (17)
C14—C15—H15A	119.8	C3—P—C23	113.70 (16)
C16—C15—H15A	119.8	C17—P—C23	107.82 (15)
C15—C16—C11	119.8 (4)	C11—P—C23	106.52 (16)
C15—C16—H16A	120.1		
			
O1—C1—C2—O2	−1.9 (6)	C25—C26—C27—C28	−0.1 (6)
N1—C1—C2—O2	176.3 (3)	C24—C23—C28—C27	0.0 (5)
O1—C1—C2—C3	179.0 (4)	P—C23—C28—C27	178.5 (3)
N1—C1—C2—C3	−2.7 (4)	C26—C27—C28—C23	0.2 (6)
O2—C2—C3—C4	−176.2 (4)	O1—C1—N1—C4	179.9 (4)
C1—C2—C3—C4	2.7 (4)	C2—C1—N1—C4	1.7 (4)
O2—C2—C3—P	−2.9 (6)	O1—C1—N1—C5	1.0 (6)
C1—C2—C3—P	176.0 (3)	C2—C1—N1—C5	−177.3 (3)
C2—C3—C4—O3	177.3 (4)	O3—C4—N1—C1	−179.2 (3)
P—C3—C4—O3	3.5 (5)	C3—C4—N1—C1	−0.1 (4)
C2—C3—C4—N1	−1.7 (4)	O3—C4—N1—C5	−0.2 (5)
P—C3—C4—N1	−175.5 (2)	C3—C4—N1—C5	178.9 (3)
C10—C5—C6—C7	1.1 (6)	C6—C5—N1—C1	123.4 (4)
N1—C5—C6—C7	−176.6 (3)	C10—C5—N1—C1	−54.3 (5)
C5—C6—C7—C8	−0.3 (6)	C6—C5—N1—C4	−55.5 (5)
C6—C7—C8—C9	−0.4 (7)	C10—C5—N1—C4	126.8 (4)
C7—C8—C9—C10	0.3 (7)	C2—C3—P—C17	−119.4 (3)
C8—C9—C10—C5	0.5 (6)	C4—C3—P—C17	53.1 (3)
C6—C5—C10—C9	−1.2 (6)	C2—C3—P—C11	0.4 (4)
N1—C5—C10—C9	176.5 (3)	C4—C3—P—C11	172.9 (3)
C16—C11—C12—C13	0.4 (6)	C2—C3—P—C23	118.4 (3)
P—C11—C12—C13	178.4 (4)	C4—C3—P—C23	−69.1 (3)
C11—C12—C13—C14	−0.9 (8)	C18—C17—P—C3	9.6 (4)
C12—C13—C14—C15	1.3 (9)	C22—C17—P—C3	−172.3 (3)
C13—C14—C15—C16	−1.1 (8)	C18—C17—P—C11	−109.7 (3)
C14—C15—C16—C11	0.5 (7)	C22—C17—P—C11	68.4 (3)
C12—C11—C16—C15	−0.1 (6)	C18—C17—P—C23	135.1 (3)
P—C11—C16—C15	−178.3 (3)	C22—C17—P—C23	−46.8 (3)
C22—C17—C18—C19	1.5 (6)	C12—C11—P—C3	−110.7 (3)
P—C17—C18—C19	179.6 (3)	C16—C11—P—C3	67.4 (3)
C17—C18—C19—C20	−2.7 (7)	C12—C11—P—C17	10.7 (4)
C18—C19—C20—C21	2.2 (8)	C16—C11—P—C17	−171.2 (3)
C19—C20—C21—C22	−0.4 (7)	C12—C11—P—C23	126.7 (3)
C20—C21—C22—C17	−0.9 (7)	C16—C11—P—C23	−55.2 (3)
C18—C17—C22—C21	0.3 (6)	C28—C23—P—C3	29.6 (3)
P—C17—C22—C21	−177.8 (3)	C24—C23—P—C3	−151.9 (3)
C28—C23—C24—C25	−0.3 (5)	C28—C23—P—C17	−94.7 (3)
P—C23—C24—C25	−178.8 (3)	C24—C23—P—C17	83.8 (3)
C23—C24—C25—C26	0.3 (6)	C28—C23—P—C11	148.6 (3)
C24—C25—C26—C27	−0.1 (6)	C24—C23—P—C11	−32.9 (3)
